# Dosimetric Comparison of Radiation Techniques for Comprehensive Regional Nodal Radiation Therapy for Left-Sided Breast Cancer: A Treatment Planning Study

**DOI:** 10.3389/fonc.2021.645328

**Published:** 2021-04-12

**Authors:** Heejoo Ko, Jee Suk Chang, Jin Young Moon, Won Hee Lee, Chirag Shah, Jin Sup (Andy) Shim, Min Cheol Han, Jong Geol Baek, Ryeong Hwang Park, Yong Bae Kim, Jin Sung Kim

**Affiliations:** ^1^ College of Medicine, The Catholic University of Korea, Seoul, South Korea; ^2^ Department of Radiation Oncology, Yonsei Cancer Center, Yonsei University College of Medicine, Seoul, South Korea; ^3^ Department of Radiation Oncology, Taussig Cancer Institute, Cleveland Clinic, Cleveland, OH, United States; ^4^ New York Proton Center, New York, NY, United States

**Keywords:** breast neoplasms, continuous positive airway pressure, proton therapy, radiotherapy, intensity-modulated

## Abstract

**Purpose:**

How modern cardiac sparing techniques and beam delivery systems using advanced x-ray and proton beam therapy (PBT) can reduce incidental radiation exposure doses to cardiac and pulmonary organs individually or in any combination is poorly investigated.

**Methods:**

Among 15 patients with left-sided breast cancer, partial wide tangential 3D-conformal radiotherapy (3DCRT) delivered in conventional fractionation (CF) or hypofractionated (HF) schedules; PBT delivered in a CF schedule; and volumetric modulated arc therapy (VMAT) delivered in an HF schedule, each under continuous positive airway pressure (CPAP) and free-breathing (FB) conditions, were examined. Target volume coverage and doses to organs-at-risk (OARs) were calculated for each technique. Outcomes were compared with one-way analysis of variance and the Bonferroni test, with *p*-values <0.05 considered significant.

**Results:**

Target volume coverage was within acceptable levels in all interventions, except for the internal mammary lymph node D95 (99% in PBT, 90% in VMAT-CPAP, 84% in VMAT-FB, and 74% in 3DCRT). The mean heart dose (MHD) was the lowest in PBT (<1 Gy) and VMAT-CPAP (2.2 Gy) and the highest in 3DCRT with CF/FB (7.8 Gy), respectively. The mean lung dose (MLD) was the highest in 3DCRT-CF-FB (20 Gy) and the lowest in both VMAT-HF-CPAP and PBT (approximately 5–6 Gy). VMAT-HF-CPAP and PBT delivered a comparable maximum dose to the left ascending artery (7.2 and 6.13 Gy, respectively).

**Conclusions:**

Both proton and VMAT in combination with CPAP can minimize the radiation exposure to heart and lung with optimal target coverage in regional RT for left-sided breast cancer. The clinical relevance of these differences is yet to be elucidated. Continued efforts are needed to minimize radiation exposures during RT treatment to maximize its therapeutic index.

## Introduction

Modern multi-disciplinary treatment for breast cancer, including radiation therapy (RT), has improved patients’ long-term survival rates (1). Concurrently, delayed side effects associated with RT are gaining more importance (2–4). Recent studies have reported that the risk of secondary lung cancer and cardiovascular toxicity is associated with the mean radiation dose delivered to the heart and lung (5). According to Darby et al., the risk of major coronary events increases linearly by 7.4% per 1-Gy increase in the mean heart dose (MHD) (6). The use of comprehensive regional nodal irradiation (RNI) in RT has increased after evidence from landmark trials became available (7, 8). However, extended RT increases the MHD, thus increasing the risk of major coronary events (9).

Recently, RT techniques such as deep inspiration breath hold (DIBH), prone RT, cardiac blocking, and continuous positive airway pressure (CPAP)—a new alternative to the DIBH technique—have been introduced to help reduce the MHD (10, 11). Fixed-beam intensity-modulated radiotherapy (IMRT), volumetric modulated arc therapy (VMAT), and proton beam therapy (PBT) can also help reduce the MHD. In light of the accumulating evidence regarding the safety and effectiveness of hypofractionated radiotherapy (HF-RT), a 3-week schedule of 40 Gy in 15 fractions is being increasingly selected in the treatment of breast cancer, especially during the current coronavirus disease (COVID-19) pandemic (12). Rotational IMRT, such as VMAT, has been suggested to reduce the dose to cardiac substructures in the hypofractionated (HF) group (13). However, how cardiac sparing techniques, IMRT/PBT, and HF-RT affect dose avoidance to the organs-at-risk (OARs) individually or in any combination is poorly understood.

This study aimed to compare target volume coverage and dose to OARs, including cardiac and pulmonary doses, in eight combinations of different RT techniques and dose schedules [three-dimensional conformal RT (3DCRT), VMAT, and PBT, with and without CPAP, delivered in a conventional fractionation (CF) or HF schedule] to establish an optimal technique for RNI in the modern era.

## Materials and Methods

Following approval by the Institutional Review Board of Yonsei University Hospital (No. 4-2020-1163), 15 patients with left-sided breast cancer treated with regional nodal RT between April and May 2020 were included. The requirement for informed consent was waived owing to the study’s retrospective nature. Three patients had undergone mastectomy without reconstruction, while the remaining patients had undergone lumpectomy. DIBH is associated with some practical challenges (such as prolonged daily treatment time and the requirement of a patient’s cooperation with high compliance). Therefore, we have used CPAP as an alternative to DIBH since 2020 (14).

All patients underwent computed tomography (CT) with free-breathing (FB) and CPAP. During RT simulation, CPAP was gradually increased to the highest level tolerated by the patient. Clinical target volumes (CTVs) for the breast and thoracic wall were delineated for each CT image, utilizing the ventral side of the major pectoral muscle, skin, and the medial mammary branches of the internal thoracic artery as borders.

CTVs of the breast axillary lymph nodes (AXL1, AXL2, and AXL3), supraclavicular lymph nodes (SCLs), and internal mammary lymph nodes (IMNs) were also delineated separately. The extent of RNI was modified at the physician’s discretion. AXL1 or AXL2 were spared for seven patients. Planning target volumes (PTVs) were generated with non-uniform margins of 3–5-mm based on CTVs, maintaining a minimum distance of 3–5 mm from the skin and lungs. OARs, including the heart, left anterior descending artery (LAD), ipsilateral lung, contralateral breast, skin, and esophagus were delineated.

Eight plans were generated for each patient: 3DCRT in CF and HF schedules, VMAT in an HF schedule, and PBT in a CF schedule, using FB- and CPAP-based CT scans. Moreover, 3DCRT in a CF schedule was planned at a dose of 50.0 Gy in 25 fractions, followed by 5 fractions of sequential boost irradiation at 2.0 Gy per fraction. Meanwhile, 3DCRT in an HF schedule was set to 40.05 Gy in 15 fractions, followed by 5 fractions of sequential boost irradiation at 2.0 Gy per fraction. VMAT in an HF schedule was planned as 40.05 Gy in 15 fractions, with simultaneous integral boost (SIB) to targets at 7.95 Gy (a total of 48.0 Gy for boost volume). PBT in CF was planned as 25 fractions of 50.0 Gy (considering the relative biological effectiveness), with 5 fractions of sequential boost irradiation at 2.0 Gy per fraction added for boost-prescribed patients. Based on our previous dosimetric study, a partially wide tangential field (PWTF) was selected and generated in the Pinnacle system as the representative of 3DCRT in this study (15). Medial and lateral 6-MV tangential fields were used to target the breast and IMN. The dose fields were normalized in the same way as the three-field technique photon field. A 6-MV dose was normalized at a point of the central axis of the fields. If any part of the heart was included in the tangential fields, a multileaf collimator was used to shield it from the photon fields. The humeral head, larynx, and trachea were also shielded by the multileaf collimator.

For VMAT, double coplanar arc plans were generated on RayStation (RayStation 5.0.3.17, RaySearch Laboratories AB, Stockholm, Sweden). A linear accelerator with 6-MV photon beams (Versa HD, Elekta, Sweden) was used in this study. Arcs 1 and 2 were simultaneously rotated in the reverse direction. In most cases, Arc 1 started at 295–305° and stopped at 155–165°. All plans were normalized to ensure that at least 95% of the chest wall PTV received 95% of the prescribed dose. The planning criteria for PTV coverage and dose to normal organs were based on institutional practice guidelines. To minimize the occurrence of hotspots, particularly in the CTV, the maximum dose to any point was limited to 105%. RayStation was used for dose calculation and optimization, in a dose grid sized 2 mm with a collapsed cone algorithm.

Dose constraints followed Danish Breast Cancer Cooperative Group guidelines and Radiation Therapy Oncology Group 1005 protocol for 3DCRT and HF-VMAT, respectively. However, a modified protocol constructed from previous studies in our institution was used. Doses to normal tissues were limited as follows: an MHD of <5 Gy; a maximum and mean dose to the LAD of <12 Gy and <5 Gy, respectively; <50%, <35%, and <20% for the ipsilateral lung volume receiving over 5 Gy (V5Gy), 10 Gy (V10Gy), and 20 Gy (V20Gy), respectively; and a mean contralateral lung dose of <2 to 3 Gy. Moreover, wherever possible, doses to the LAD, heart, and other normal tissues were kept as low as possible while ensuring PTV coverage. PBT plans were generated using the pencil beam scanning technique in Raystation9A (RaySearch Laboratories AB). Two beams, including one anterior beam and one left oblique beam at an angle ranging from 30° to 45°, were used. For PBT plans, the area 5 mm inside the boundary of the external contour was excluded from PTVs to spare the skin, and a margin of 1 mm was added with respect to the specific distal and proximal beams. Isotropic 5-mm uncertainty and 3.5% range uncertainty were accounted for in the robust optimization algorithm. Dose criteria were set based on a combination of the Radiotherapy Comparative Effectiveness Consortium Trial (RADCOMP) description and clinical guidelines of the New York Proton Center. At least 95% of each PTV was expected to receive >95% of the prescribed dose. The maximum dose to the PTV was restricted to 110% of the prescribed dose. The MHD was set as <15 Gy and ipsilateral lung V20Gy was set as <50% to comply with the mandatory constraints criteria of RADCOMP (16). However, wherever possible, an ipsilateral lung V20Gy of <15%, MHD of <2 Gy, maximum dose to the heart surface of <20 Gy, and maximum dose to the skin of <95% of the prescribed dose were used as planning objectives.

Target volume coverage and doses to OARs were calculated for each RT technique. Target volume coverage was evaluated with D90 (dose to 90% of the target volume), D95 (dose to 95% of the target volume), homogeneity index (HI), and conformity index (CI). D90 and D95 were reported as the percentage of the prescribed dose, as the prescribed doses differed between techniques. Composite plans with individual PTVs for the whole breast, boost, AXL nodes, SCLs, and IMNs were evaluated separately for each target volume. Doses to the heart, LAD, ipsilateral lung, contralateral breast, and skin were calculated for dose evaluation for OARs. The 5-mm zone inside the boundary of the external contour was considered the skin area. All 120 plans met the mandatory constraint criteria for PTVs and OARs, as described. The resulting parameter estimates were compared using one-way analysis of variance and the Bonferroni test using R (v.4.0.2, The R Foundation for Statistical Computing, Vienna, Austria) in RStudio (v.1.3, RStudio Inc., Boston, MA). *p*-values of <0.05 were considered indicative of statistical significance.

## Results

**Figure 1:**
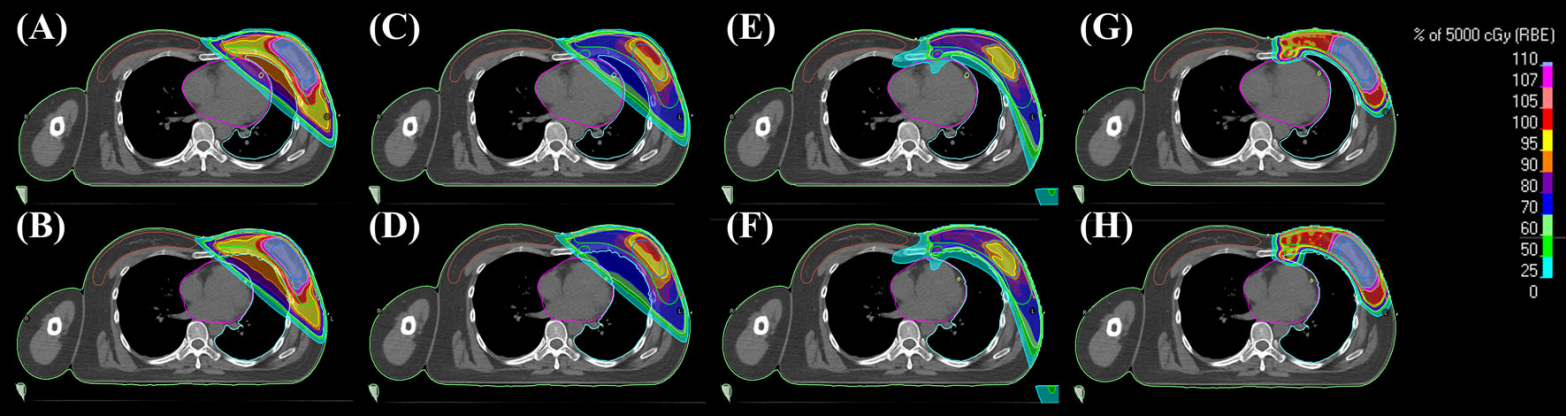
Representative 2D dose distributions in a patient. **(A)** 3DCRT-FB-CF, **(B)** 3DCRT-CPAP-CF, **(C)** 3DCRT-FB-HF, **(D)** 3DCRT-CPAP-HF, **(E)** VMAT-FB-HF, **(F)** VMAT-CPAP-HF, **(G)** PBT-FB-CF, and **(H)** PBT-CPAP-CF on the same level cut of CT images. 3DCRT, three-dimensional conformal radiotherapy; VMAT, volumetric modulated arc therapy; PBT, proton beam therapy; CPAP, continuous positive airway pressure; CF, conventional fractionation; HF, hypofractionation.

**Figure 2:**
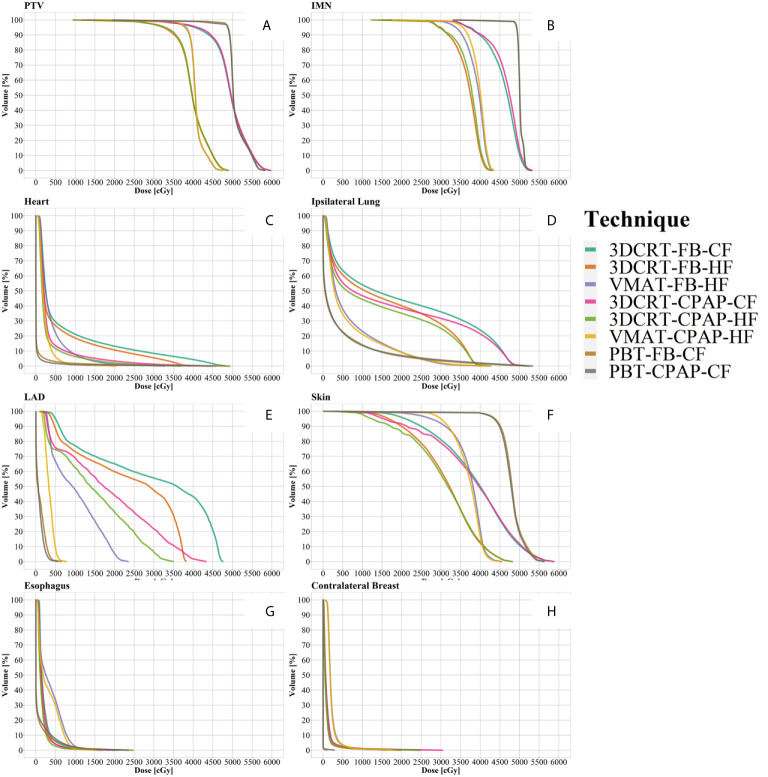
Dose-volume histograms (DVH). **(A)** PTV, **(B)** IMN, **(C)** heart, **(D)** ipsilateral lung, **(E)** LAD, **(F)** skin, **(G)** esophagus, and **(H)** contralateral breast. Lines represent the mean DVH curve of each technique. IMN, internal mammary lymph nodes; LAD, left anterior descending artery; PTV, planning target volume.

Representative cross-sectional dose distributions of all eight techniques in a single patient on the same slice of CT images are illustrated in **Figure 1**. **Figure 2** depicts the mean dose-volume histograms of 15 patients for each of the eight techniques with respect to key PTVs and OARs. The mean values of PTV coverage and OARs of interest in 15 patients are presented in **Supplementary Table 1**.

### PTV

**Figure 3:**
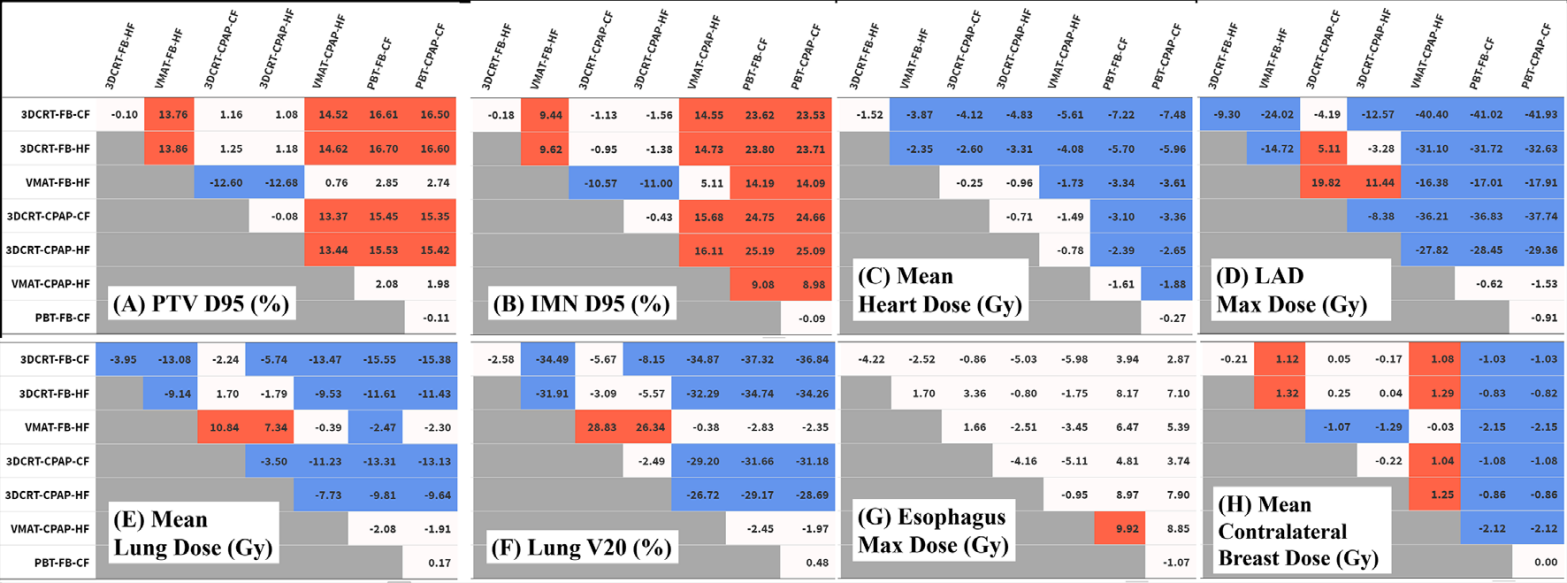
Differences in dose distribution values. Each table represents the difference between techniques in the values of **(A)** PTV D95 (%), **(B)** IMN D95 (%), **(C)** mean heart dose (Gy), **(D)** LAD maximum dose (Gy), **(E)** mean lung dose (Gy), **(F)** lung V20 (%), **(G)** esophagus V20 (%), and **(H)** mean contralateral breast dose (Gy). Numeric values represent the difference of the value for the technique in the column from the technique in the row. Red cells indicate significant positive differences and blue cells indicate significant negative differences. IMN, internal mammary lymph nodes; LAD, left anterior descending artery; PTV, planning target volume.

Regarding PTV coverage, PBT and VMAT were superior in terms of D90, D95, HI, and CI to 3DCRT, with either an HF or CF schedule. There was no notable difference in boost coverage between any techniques. In contrast to coverage for the other nodes, which followed the tendency observed for whole PTV coverage, PBT achieved greater coverage of IMNs than VMAT, with statistical significance (in D90 and D95). No significant difference between the use of CPAP and FB was observed in any PTV coverage results (**Figures 3A, B** **)**.

### Heart

The MHD values varied considerably between techniques. PBT achieved the lowest MHD of 0.47 Gy. The change in MHD with the use of CPAP in PBT was non-significant. Except for PBT, the use of CPAP significantly reduced MHD in every technique in comparison with corresponding FB conditions. In VMAT with CPAP, the mean MHD was 2.22 Gy, which was 44% lower than the corresponding value achieved with VMAT in FB conditions (3.95 Gy) and which was non-inferior to that achieved with PBT in FB conditions (**Figure 3C**).

### LAD

Five techniques, including all four 3DCRT techniques and VMAT in FB conditions, showed maximum LAD doses of >20 Gy, while the other three techniques achieved doses of <10 Gy. Among photon-based techniques, only VMAT with CPAP achieved the maximum LAD dose that was non-inferior to that achieved by PBT (**Figure 3D**).

### Ipsilateral Lung

VMAT and PBT techniques significantly reduced the mean ipsilateral lung dose (MLD) compared with that achieved with 3DCRT. Regarding MLD, no significant difference was found with the use of CPAP, in comparison to the use of relevant RT techniques with FB. PBT attained the lowest MLD (4.49 Gy), while VMAT-based techniques achieved an MLD of 6.67 Gy (**Figure 3E**).

**Figure 4:**
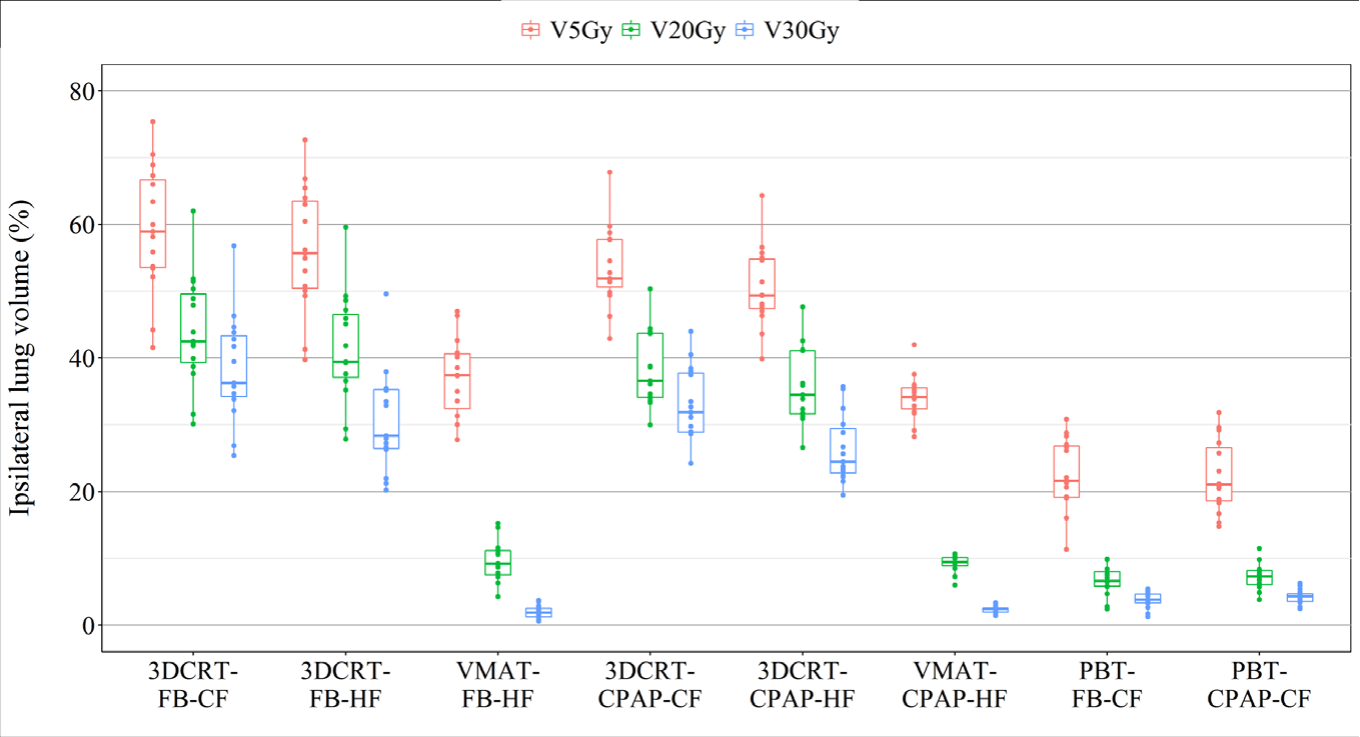
Ipsilateral lung volumes receiving over 5, 20, and 30 Gy per patient per technique.

The percentage of the ipsilateral lung volume receiving over 20 Gy (V20) was lower in VMAT and PBT than in 3DCRT. The corresponding values were comparable between VMAT and PBT techniques (approximately 7–9%) (**Figure 3F**). **Figure 4** presents the estimates of the lowest ipsilateral lung V5 and V10 (volume receiving >5 Gy and >10 Gy, respectively) achieved with PBT techniques.

### Skin

The dose administered to 1% of the volume of the skin (D1%) was approximately 108–109% in VMAT and PBT, compared to >112% in 3DCRT. However, there were no significant differences between techniques.

### Esophagus

Regarding the esophagus, there was no significant difference in V20 between techniques. The maximum esophageal dose was not significantly different between photon techniques, and VMAT with CPAP resulted in a dose 9.9 Gy lower than PBT in FB. In terms of mean esophageal dose, PBT achieved the lowest dose of 1 Gy and VMAT showed the highest with 3.4 Gy (**Figure 3G**).

### Contralateral Breast

**Figure 5:**
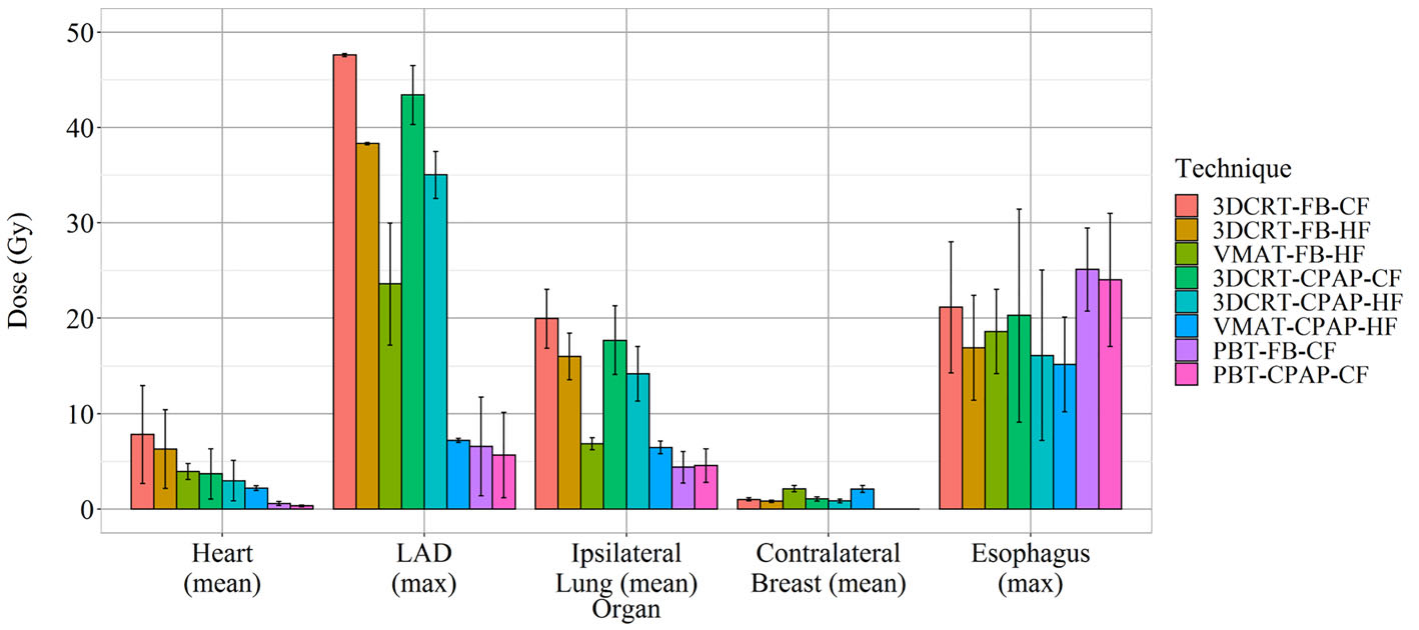
Doses to organs-at-risk per technique, aimed at decreasing the mean heart dose.

VMAT techniques achieved a mean contralateral breast dose of >2.1 Gy, which was greater than those achieved by the other techniques. In contrast, PBT techniques achieved a mean contralateral breast dose of <0.005 Gy, which was lower than those achieved by the other techniques (**Figure 3H**). Findings for the OARs are summarized in **Figure 5**.

## Discussion

### Heart-Sparing Efforts in the Era of RNI

Disease-free survival in patients with breast cancer has improved by 3–5%, according to the results of modern clinical trials involving RNI, including RNI of SCLs, AXLs, and IMNs (7, 8). Maximizing the therapeutic efficacy of RNI requires the improvement of the accuracy of dose delivery to the target volumes, while sparing the surrounding OARs, which may be achieved with modern techniques used individually or in combination. In this study, we compared three RT techniques (standard 3D photon, VMAT, and PBT) with or without CPAP delivered in CF or HF schedules to 15 patients with left-sided breast cancer whose regional nodes were being treated.

IMRT has been proposed as a heart-sparing technique, although findings regarding MHD have been conflicting. In a recent prospective study from the Memorial Sloan-Kettering Cancer Center (17), an MHD of 13.2 Gy (range, 8.6–20 Gy) was reported in patients with left-sided breast cancer who received multibeam IMRT. In a Korean dummy-run study (KROG 1901), 21 institutions received a representative case and were requested to create a complete RT plan from target delineation according to respective institutional protocols (18). Although all submitted plans were generated for IMRT (13 fixed-field IMRT, 7 VMAT, and 1 helical tomotherapy), the median MHD was 12.5 Gy with a wide range (3.3–24.1 Gy). In a study by Pham et al., an MHD of 5.7 Gy was reported with VMAT-DIBH (19).

IMRT techniques vary in sophistication, and discrepancies in MHD can be accounted for by several factors. These factors include differences in methods used for the balancing of target volume coverage versus the minimization of MHD in the inverse planning process, the use of the DIBH technique or CPAP, the use of different fractionation schedules, and inter-physician/institution variation in target delineation and PTV setup margins. Quality assurance schemes for RT plans and regular audits with peer review might help establish best-practice guidelines for the use of IMRT in breast cancer RT.

HF schedules have become a new standard in breast cancer RT, particularly in patients with early-stage disease. Although evidence from a recent randomized trial in China supported the use of HF schedules in the treatment of locally advanced breast cancer (20), concerns exist over the safety and efficacy profile of HF in RNI settings (21). For example, the 40/15 regimen, used in the present study, is a popular regimen in RNI settings in the United Kingdom and Korea and was endorsed by the guidelines issued during the COVID-19 pandemic (12). Moreover, it is currently being tested in clinical trials in Denmark and France (NCT02384733 and NCT03127995). Concurrently, in North America, an HF regimen of >40/15 in 2-Gy equivalent doses is being tested in randomized trials (Alliance A221505 [NCT03414970] and FABREC [NCT03422003]).

### 3DCRT-FB and PBT at the Opposite Ends

In the present study, 3DCRT (PWTF in this study) in FB conditions, as the representative conventional treatment, showed the following dosimetry profile: the D95 of the PTV was 81% of the prescribed dose, with a CI of 0.52, skin D1% of 113%, maximum LAD dose of 48 Gy, and MLD of 20 Gy. The MHD of 7.8 Gy was slightly lower than that previously reported yet consistent with that presented in studies using wide tangents or matched photon-electron fields in FB conditions (approximately 9 Gy) (15, 22). Meanwhile, the discrepancy in findings may be explained by the reduced coverage of IMN target volumes due to partial heart block with a PWTF in the current study (D95 75%, D90 80%).

In the present study, PBT was associated with the lowest doses to most OARs and with the best target volume coverage: the D95 of the PTV was 98% of the prescribed dose, with a CI of 0.92, IMN coverage (D95) of 99%, skin D1% of 109%, maximum LAD dose of 6.1 Gy, MLD of 4.5 Gy, and MHD of 0.5 Gy, which were consistent with or slightly better than the values previously reported for PBT (23, 24). This might be due to the use of two beams, the skin being clipped out of the PTV, or active OAR-sparing during RT planning. Nevertheless, the clinical benefit of this excellent dose distribution remains unclear and subject to scrutiny in multi-center randomized trials based in North America (RADCOMP; NCT02603341) and Denmark (The DBCG Proton; NCT04291378).

Standard 3DCRT under FB conditions and PBT are at the opposite ends of the spectrum in terms of MHD. However, it is important to assess the impact of modern photon RT techniques and their relative contributions to any increase in the risk of long-term side effects, as proton beams are not universally accessible or cost-effective in the treatment of women without comorbidities or of those who receive an MHD of <5 Gy (25). The DBCG proton trial is recruiting patients with indications for RT, wherein standard RT planning reveals an MHD of >4 Gy.

### VMAT With CPAP in HF: An Encouraging Choice

The dosimetric profile of VMAT with CPAP in an HF schedule reported in the present study is encouraging: the D95 of the PTV was 96% of the prescribed dose, with a CI of 0.91, IMN coverage (D95) of 90%, skin D1% of 107%, maximum LAD dose of 7.2 Gy, left lung V20 of 9%, and MHD of 2.2 Gy. In the FB condition, a 50% reduction in MHD from 7.8 Gy (3DCRT) to 3.9 Gy was achieved with the use of VMAT. These values are similar to those previously reported for VMAT with the DIBH technique in a study in the United Kingdom (PTV nodes 96% and MHD 2.6 Gy), with the exception of the maximum LAD dose (23.3 Gy) and left lung dose (V17Gy 28%) (24).

Evidence suggests that the MHD can be reduced by 50% with the use of the DIBH technique (26, 27). In the present study, we observed a 53% reduction in the MHD due to the use of CPAP (MHD of 3DCRT with CPAP compared with those in 3DCRT under FB conditions in either CF or HF schedules); this finding was consistent with those of previous studies. In the present study, the use of VMAT with CPAP achieved an additional 40.1% reduction in the MHD (compared with those with 3DCRT with CPAP in CF, from 3.7 Gy to 2.2 Gy). Compared with PBT in FB, only VMAT with CPAP showed no significant difference in MHD among photon techniques, which indicates that the gap between photon and proton RT has narrowed substantially with modern RT techniques.

Some observations in results were statistically non-significant but notable. An MHD of 1.77 Gy was observed in VMAT with CPAP at a pressure of 17 cmH_2_O (versus 2.6 Gy and 2.3 Gy achieved at pressures of 15 cmH_2_O and 12 cmH_2_O, respectively). Furthermore, ipsilateral lung V20 was reduced to 9% in VMAT with CPAP, a result comparable with that of lung-sparing PBT.

In the present study, as a consequence of using the 40/15 regimen instead of the 50/25 regimen, albeit not statistically significant, the MHD decreased by 1.5 Gy and 0.71 Gy under FB and CPAP conditions, respectively. The maximum LAD dose showed a significant reduction of 9.3 Gy and 8.4 Gy with an HF schedule in FB and CPAP conditions, respectively; this finding is similar to that reported by Pierre et al. (13). In addition, the 40/15 HF schedule can help reduce the lung dose to a greater extent than the 50/25 schedule, a result consistent with that of a previous study (28). However, the clinical relevance of these dosimetric differences associated with HF regimens is yet to be elucidated.

### Other OARs

Regarding the contralateral breast dose, the present findings are consistent with those of previous studies (24). Only PBT techniques were able to spare the contralateral breast tissue, delivering a dose close to 0 Gy (0.003–0.005 Gy). Although VMAT significantly increased the dose delivered to the contralateral breast compared with that delivered by 3DCRT, the mean dose difference between these techniques was approximately 1 Gy. According to a recent study based on the National Cancer Database, which includes data regarding breast cancer and other tumor types, the relative risk of secondary cancer associated with IMRT is similar to that associated with 3DCRT (29), suggesting that these dose differences may not translate to clinically relevant outcomes in most middle-age and older women.

The esophageal dose was comparable between VMAT and 3DCRT, and significantly lower in photon techniques compared to PBT. Our findings with respect to proton versus photon are similar to a recent study by Paganetti et al. (30). However, our findings with respect to VMAT versus 3DCRT contradicts previous studies. In one study where the esophagus was not contoured (31) and another study where the dose-constraint of the esophagus (Dmax less than 40 Gy) was higher than our method (D0.03cc less than 12 Gy) (30), the esophageal dose was higher in patients treated with IMRT compared to patients treated with 3DCRT. A study by Yaney et al. also showed that these dosimetric differences translated into an increased rate of grade 2 esophagitis in patients with IMRT.

### Personalized RT Strategies

Overall, these findings suggest that each of the eight techniques can achieve adequate target volume coverage with varying doses delivered to OARs, indicating that RT for breast cancer should be personalized based on each patient’s anatomical characteristics. Bazan et al. recently reported the potential of an adaptive treatment planning algorithm for IMRT versus 3DCRT in RNI settings (32). In the algorithm, IMRT was used in 30% of patients for whom 3DCRT did not meet the critical OAR constraint criteria. Moreover, Hytonen et al. recently presented the feasibility of an automated patient-specific evidence-based decision-making system for optimizing proton or photon treatment based on normal tissue complication probability (18). In a simulation of this study, PBT was indicated for 22% of the patients, and multiple patients were close to the decision threshold at the same time. Taken together, the present findings may contribute to the development of treatment strategies for patients with left-sided breast cancer undergoing RNI.

### Limitations

PBT is not universally accessible and CPAP for breast cancer RT is presently a novel technique. Since non-contrast CT images were used in this study, uncertainties in the contouring of LAD vessels might exist. Moreover, it was not possible to delineate the left ventricle separately on non-contrast CT, and thus we were unable to evaluate doses to the cardiac substructures (such as the left ventricle receiving 5 Gy; LV-V5); therefore, the heart was considered a single organ. Nevertheless, we recently reported a significant MHD effect per Gy for cardiac toxicity, particularly in the era of CT-based individual dose calculation in women with breast cancer (33). The clinical relevance of the dosimetric differences found in this study is yet to be elucidated.

## Conclusion

Among available techniques, modern PBT can achieve the lowest doses to most OARs in the regional nodal treatment of left-sided breast cancer. The gap in the MHD between proton and photon RT has narrowed substantially with modern techniques. In VMAT-HF with CPAP, an MHD and maximum LAD dose of approximately 2 Gy and 7 Gy, respectively, were achieved with adequate target volume coverage and a reduced lung dose (V20 9%, MLD 6 Gy). The clinical relevance of these dosimetric differences is yet to be elucidated. This study provides a precise dosimetric comparison between modern breast RT techniques to assist institutions in selecting the optimal RT regimens based on their respective resources.

## Data Availability Statement

The original contributions presented in the study are included in the article/**Supplementary Material**. Further inquiries can be directed to the corresponding author.

## Ethics Statement

The studies involving human participants were reviewed and approved by Institutional Review Boards of Yonsei University Hospital (4-2020-1163). The patients/participants provided their written informed consent to participate in this study.

## Author Contributions

HK, JC, and JK conceived the study and performed all the analysis and drafted the manuscript. JM, WL, CS, JS, MH, JB, RP, and YK critically reviewed the text and contributed to the clinical analysis. All authors contributed to the article and approved the submitted version.

## Funding

This work was supported by the National Research Foundation of Korea (NRF) grant funded by the Korean government (MSIT) (2020R1A4A1016619).

## Conflict of Interest

JK is a cofounder of Oncosoft and serves as an advisor at Rayence. JC acts as a scientific consultant at Accuray.

The remaining authors declare that the research was conducted in the absence of any commercial or financial relationships that could be construed as a potential conflict of interest.
