# Supplementary material for: Dosimetric Comparison of Radiation Techniques for Comprehensive Regional Nodal Radiation Therapy for Left-Sided Breast Cancer: A Treatment Planning Study
Source: Front Oncol. 2021 Apr 12;11:645328. doi: 10.3389/fonc.2021.645328 (PMC8072050; doi:10.3389/fonc.2021.645328)

Supplementary Material

**1 Supplementary Table 1.** Mean and standard deviation of planning target volume coverage and doses to organs-at-risk for 15 patients

|  | Mean Heart Dose (Gy) | Maximum LAD Dose (Gy) | Mean Ipsilateral Lung Dose (Gy) | Ipsilateral Lung V20 (%) | Skin D1 (%) | Mean Contralateral Breast Dose (Gy) | Mean Esophagus Dose (Gy) | Maximum Esophagus Dose (Gy) |
| --- | --- | --- | --- | --- | --- | --- | --- | --- |
| 3DCRT-FB-CF | 7.82(2.66) | 47.60(3.01) | 19.95(3.69) | 44.06(8.30) | 112.52(6.03) | 1.04(0.42) | 2.13(0.53) | 21.14(12.31) |
| 3DCRT-FB-HF | 6.30(2.13) | 38.30(2.60) | 16.00(2.96) | 41.48(8.14) | 114.93(8.22) | 0.83(0.34) | 1.72(0.42) | 16.92(9.85) |
| VMAT-FB-HF | 3.95(0.83) | 23.59(6.46) | 6.87(1.10) | 9.57(3.03) | 108.64(5.07) | 2.15(0.76) | 3.65(0.80) | 18.62(6.88) |
| 3DCRT-CPAP-CF | 3.70(1.49) | 43.41(5.84) | 17.71(2.41) | 38.39(5.64) | 112.77(6.15) | 1.08(0.54) | 1.85(0.51) | 20.28(11.62) |
| 3DCRT-CPAP-HF | 2.99(1.20) | 35.03(4.71) | 14.21(1.95) | 35.91(5.69) | 115.14(8.19) | 0.87(0.43) | 1.49(0.41) | 16.12(9.30) |
| VMAT-CPAP-HF | 2.22(0.44) | 7.20(1.02) | 6.48(0.57) | 9.19(1.37) | 107.45(4.09) | 2.12(0.67) | 3.16(0.52) | 15.17(2.89) |
| PBT-FB-CF | 0.61(0.30) | 6.58(4.15) | 4.40(1.14) | 6.74(2.29) | 109.33(8.78) | 0.00(0.01) | 0.93(0.50) | 25.09(4.73) |
| PBF-CPAP-CF | 0.34(0.19) | 5.67(3.07) | 4.57(0.94) | 7.22(1.90) | 108.80(8.51) | 0.01(0.01) | 1.05(0.49) | 24.02(4.98) |
|  | **PTV Coverage** | | | | **IMN Coverage** | | | |
|  | D90 (%) | D95 (%) | HI | CI | D90 (%) | D95 (%) | HI | CI |
| 3DCRT-FB-CF | 88.03(4.81) | 81.14(8.35) | 0.72(0.08) | 0.52(0.13) | 79.69(6.99) | 75.00(7.37) | 0.74(0.07) | 0.01(0.01) |
| 3DCRT-FB-HF | 88.05(4.89) | 81.04(8.34) | 0.71(0.09) | 0.51(0.12) | 79.54(7.04) | 74.82(7.41) | 0.73(0.07) | 0.01(0.01) |
| VMAT-FB-HF | 97.52(0.79) | 94.90(1.35) | 0.86(0.06) | 0.91(0.05) | 88.47(5.20) | 84.44(6.35) | 0.81(0.07) | 0.02(0.01) |
| 3DCRT-CPAP-CF | 89.03(4.65) | 82.30(6.15) | 0.73(0.06) | 0.52(0.12) | 80.74(6.54) | 73.88(9.58) | 0.72(0.09) | 0.01(0.01) |
| 3DCRT-CPAP-HF | 88.95(4.60) | 82.22(6.07) | 0.72(0.07) | 0.51(0.11) | 81.21(6.36) | 73.44(9.36) | 0.72(0.09) | 0.01(0.01) |
| VMAT-CPAP-HF | 97.81(0.61) | 95.66(0.88) | 0.86(0.06) | 0.91(0.04) | 92.66(1.81) | 89.55(2.74) | 0.85(0.04) | 0.02(0.01) |
| PBT-FB-CF | 98.77(0.64) | 97.75(0.61) | 0.88(0.07) | 0.92(0.04) | 99.08(0.24) | 98.63(0.41) | 0.96(0.04) | 0.03(0.01) |
| PBF-CPAP-CF | 98.69(0.70) | 97.64(0.62) | 0.88(0.07) | 0.92(0.04) | 99.05(0.25) | 98.53(0.44) | 0.96(0.04) | 0.03(0.02) |

**2 Sensitivity Analysis**

Sensitivity analysis according to surgery type was conducted to see whether surgery type would influence the results. The results appear to be similar by surgery type.

**2.1 Supplementary Table 2.** Mean and standard deviation of planning target volume coverage and doses to organs-at-risk for patients who have undergone each surgery. (A) 12 breast conservation surgery patients (B) 3 modified radical mastectomy patients

| (A) | Mean Heart Dose (Gy) | Maximum LAD Dose (Gy) | Mean Ipsilateral Lung Dose (Gy) | Ipsilateral Lung V20 (%) | Skin D1 (%) | Mean Contralateral Breast Dose (Gy) | Mean Esophagus Dose (Gy) | Maximum Esophagus Dose (Gy) |
| --- | --- | --- | --- | --- | --- | --- | --- | --- |
| 3DCRT-FB-CF | 7.64(2.01) | 47.24(3.29) | 20.54(3.70) | 45.68(8.04) | 113.48(6.29) | 1.09(0.46) | 2.24(0.54) | 22.08(13.40) |
| 3DCRT-FB-HF | 6.16(1.61) | 38.07(2.88) | 16.48(2.97) | 42.99(7.93) | 116.53(8.39) | 0.87(0.37) | 1.80(0.43) | 17.67(10.72) |
| VMAT-FB-HF | 4.03(0.85) | 23.85(6.73) | 6.98(1.18) | 9.82(3.24) | 109.50(5.35) | 2.19(0.84) | 3.75(0.80) | 18.86(7.51) |
| 3DCRT-CPAP-CF | 3.38(0.99) | 42.60(6.18) | 17.92(2.20) | 39.00(5.31) | 113.75(6.43) | 1.15(0.58) | 1.93(0.53) | 20.59(12.19) |
| 3DCRT-CPAP-HF | 2.74(0.82) | 34.46(5.04) | 14.39(1.78) | 36.51(5.18) | 116.74(8.36) | 0.92(0.47) | 1.55(0.42) | 16.34(9.76) |
| VMAT-CPAP-HF | 2.21(0.48) | 7.26(1.14) | 6.46(0.58) | 9.10(1.46) | 107.97(4.44) | 2.16(0.74) | 3.22(0.45) | 14.98(2.45) |
| PBT-FB-CF | 0.65(0.31) | 5.66(3.53) | 4.41(1.07) | 6.76(2.00) | 111.59(8.37) | 0.00(0.01) | 0.89(0.42) | 24.39(4.73) |
| PBF-CPAP-CF | 0.37(0.20) | 5.71(2.89) | 4.48(0.72) | 6.93(1.10) | 110.99(8.12) | 0.00(0.01) | 0.98(0.37) | 23.09(4.25) |
|  | **PTV Coverage** | | | | **IMN Coverage** | | | |
|  | D90 (%) | D95 (%) | HI | CI | D90 (%) | D95 (%) | HI | CI |
| 3DCRT-FB-CF | 87.37(5.13) | 79.75(8.76) | 0.70(0.07) | 0.55(0.11) | 77.69(6.03) | 73.12(6.89) | 0.72(0.06) | 0.00(0.01) |
| 3DCRT-FB-HF | 87.43(5.24) | 79.65(8.75) | 0.68(0.07) | 0.54(0.11) | 77.53(6.09) | 72.92(6.92) | 0.72(0.07) | 0.00(0.01) |
| VMAT-FB-HF | 97.67(0.71) | 95.08(1.02) | 0.84(0.06) | 0.91(0.04) | 88.31(4.96) | 84.19(5.95) | 0.80(0.07) | 0.02(0.01) |
| 3DCRT-CPAP-CF | 88.53(5.03) | 81.51(6.52) | 0.71(0.05) | 0.54(0.11) | 78.84(5.09) | 72.23(8.90) | 0.71(0.08) | 0.00(0.01) |
| 3DCRT-CPAP-HF | 88.46(4.98) | 81.44(6.42) | 0.69(0.06) | 0.54(0.11) | 79.45(5.08) | 71.71(8.52) | 0.70(0.08) | 0.00(0.01) |
| VMAT-CPAP-HF | 97.86(0.66) | 95.58(0.94) | 0.84(0.06) | 0.91(0.04) | 92.41(1.42) | 89.25(2.16) | 0.84(0.04) | 0.02(0.01) |
| PBT-FB-CF | 99.06(0.21) | 98.00(0.29) | 0.86(0.06) | 0.92(0.04) | 99.10(0.20) | 98.67(0.32) | 0.96(0.04) | 0.02(0.01) |
| PBF-CPAP-CF | 99.00(0.26) | 97.87(0.40) | 0.86(0.07) | 0.92(0.04) | 99.06(0.26) | 98.56(0.43) | 0.96(0.04) | 0.02(0.01) |

| (B) | Mean Heart Dose (Gy) | Maximum LAD Dose (Gy) | Mean Ipsilateral Lung Dose (Gy) | Ipsilateral Lung V20 (%) | Skin D1 (%) | Mean Contralateral Breast Dose (Gy) | Mean Esophagus Dose (Gy) | Maximum Esophagus Dose (Gy) |
| --- | --- | --- | --- | --- | --- | --- | --- | --- |
| 3DCRT-FB-CF | 8.56(5.14) | 49.05(0.16) | 17.60(3.06) | 37.57(6.95) | 108.67(2.98) | 0.83(0.14) | 1.71(0.21) | 17.42(6.85) |
| 3DCRT-FB-HF | 6.85(4.11) | 39.24(0.13) | 14.08(2.45) | 35.42(7.06) | 108.54(2.96) | 0.66(0.11) | 1.37(0.17) | 13.94(5.48) |
| VMAT-FB-HF | 3.63(0.83) | 22.53(6.38) | 6.41(0.63) | 8.55(2.18) | 105.20(0.26) | 2.00(0.34) | 3.23(0.78) | 17.68(4.38) |
| 3DCRT-CPAP-CF | 4.99(2.64) | 46.63(3.08) | 16.87(3.58) | 35.97(7.50) | 108.89(2.95) | 0.81(0.23) | 1.55(0.32) | 19.05(11.16) |
| 3DCRT-CPAP-HF | 3.99(2.12) | 37.31(2.46) | 13.50(2.87) | 33.48(8.23) | 108.76(2.94) | 0.65(0.18) | 1.23(0.26) | 15.24(8.92) |
| VMAT-CPAP-HF | 2.24(0.24) | 6.99(0.22) | 6.58(0.65) | 9.52(1.09) | 105.36(0.20) | 1.96(0.35) | 2.92(0.84) | 15.91(4.94) |
| PBT-FB-CF | 0.43(0.21) | 10.26(5.18) | 4.36(1.66) | 6.62(3.83) | 100.29(1.02) | 0.00(0.01) | 1.09(0.85) | 27.90(4.34) |
| PBF-CPAP-CF | 0.23(0.10) | 5.52(4.46) | 4.96(1.76) | 8.35(4.02) | 100.03(0.53) | 0.01(0.01) | 1.30(0.90) | 27.72(6.96) |
|  | **PTV Coverage** | | | | **IMN Coverage** | | | |
|  | D90 (%) | D95 (%) | HI | CI | D90 (%) | D95 (%) | HI | CI |
| 3DCRT-FB-CF | 90.66(2.04) | 86.73(2.80) | 0.82(0.04) | 0.37(0.06) | 87.67(4.76) | 82.53(3.51) | 0.79(0.05) | 0.01(0.01) |
| 3DCRT-FB-HF | 90.55(2.04) | 86.62(2.79) | 0.82(0.04) | 0.37(0.07) | 87.56(4.75) | 82.43(3.51) | 0.79(0.05) | 0.01(0.01) |
| VMAT-FB-HF | 96.91(0.98) | 94.17(2.45) | 0.91(0.03) | 0.89(0.06) | 89.11(7.29) | 85.44(9.25) | 0.82(0.10) | 0.03(0.01) |
| 3DCRT-CPAP-CF | 91.01(2.15) | 85.46(3.53) | 0.81(0.05) | 0.40(0.07) | 88.34(6.97) | 80.48(11.15) | 0.77(0.12) | 0.02(0.02) |
| 3DCRT-CPAP-HF | 90.89(2.15) | 85.35(3.52) | 0.81(0.05) | 0.40(0.07) | 88.24(6.96) | 80.38(11.14) | 0.77(0.12) | 0.02(0.02) |
| VMAT-CPAP-HF | 97.60(0.34) | 96.01(0.60) | 0.92(0.01) | 0.94(0.03) | 93.64(3.19) | 90.74(4.92) | 0.86(0.06) | 0.04(0.01) |
| PBT-FB-CF | 97.59(0.23) | 96.75(0.48) | 0.95(0.01) | 0.91(0.01) | 98.98(0.42) | 98.46(0.74) | 0.97(0.01) | 0.05(0.02) |
| PBF-CPAP-CF | 97.42(0.17) | 96.74(0.53) | 0.95(0.01) | 0.91(0.01) | 99.03(0.27) | 98.41(0.55) | 0.97(0.01) | 0.06(0.00) |

**2.2 Supplementary Figure 1.** Differences between techniques in the values of (A) PTV D95 (%), (B) IMN D95 (%), (C) mean heart dose (Gy), (D) LAD maximum dose (Gy), (E) mean lung dose (Gy), (F) lung V20 (%), (G) esophagus V20 (%), and (H) mean contralateral breast dose (Gy) for the patients who have undergone BCS and MRM each.

Numeric values represent the difference of the value for the technique in the column from the technique in the row. Red cells indicate significant positive differences and blue cells indicate significant negative differences.

IMN, internal mammary lymph nodes; LAD, left anterior descending artery; PTV, planning target volume; BCS, breast conservation surgery; MRM, modified radical mastectomy


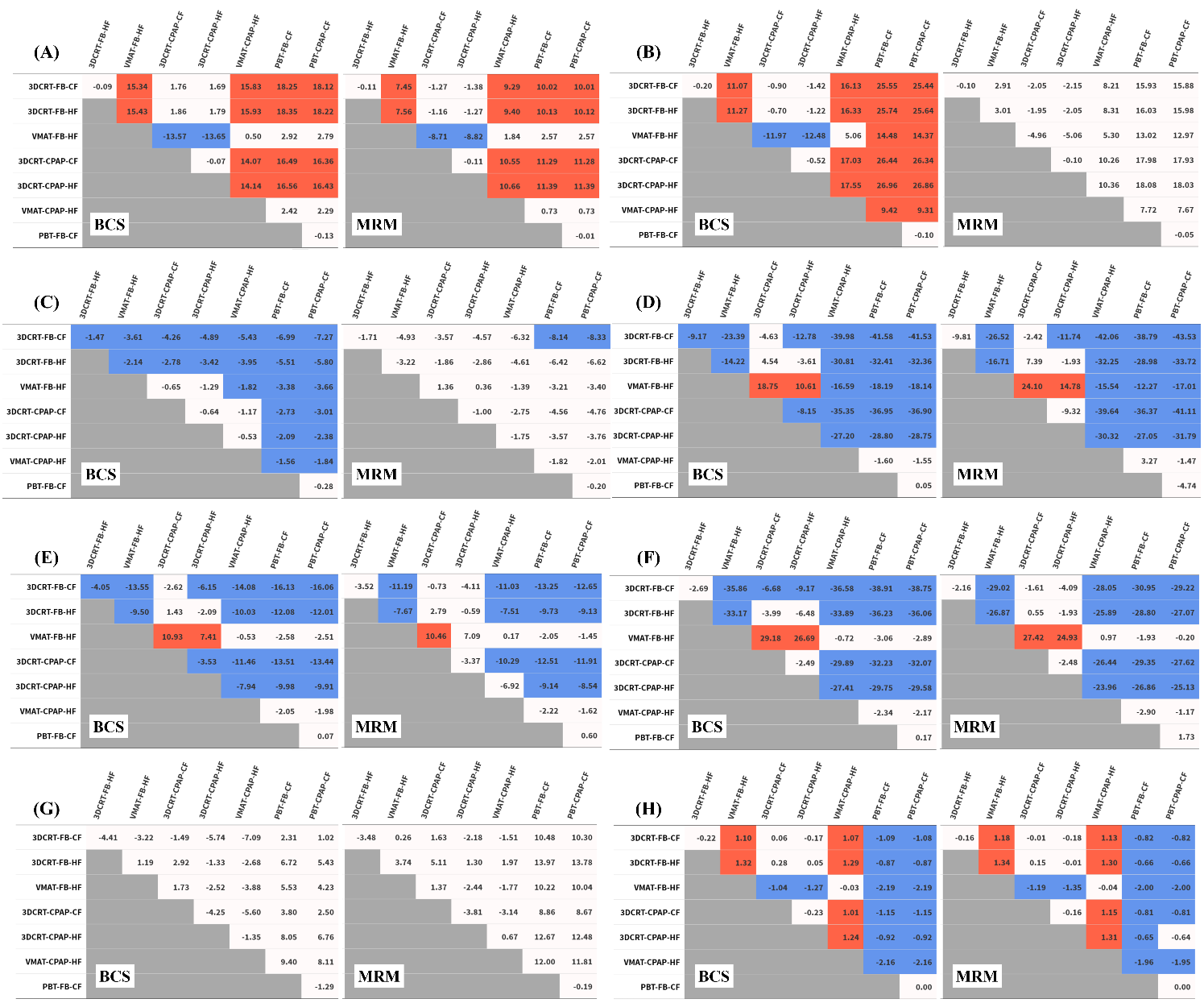


**2.3 Supplementary Figure 2.** Doses to organs-at-risk per technique, aimed at decreasing the mean heart dose for the patients who have undergone each surgery. (A) 12 breast conservation surgery patients (B) 3 modified radical mastectomy patients


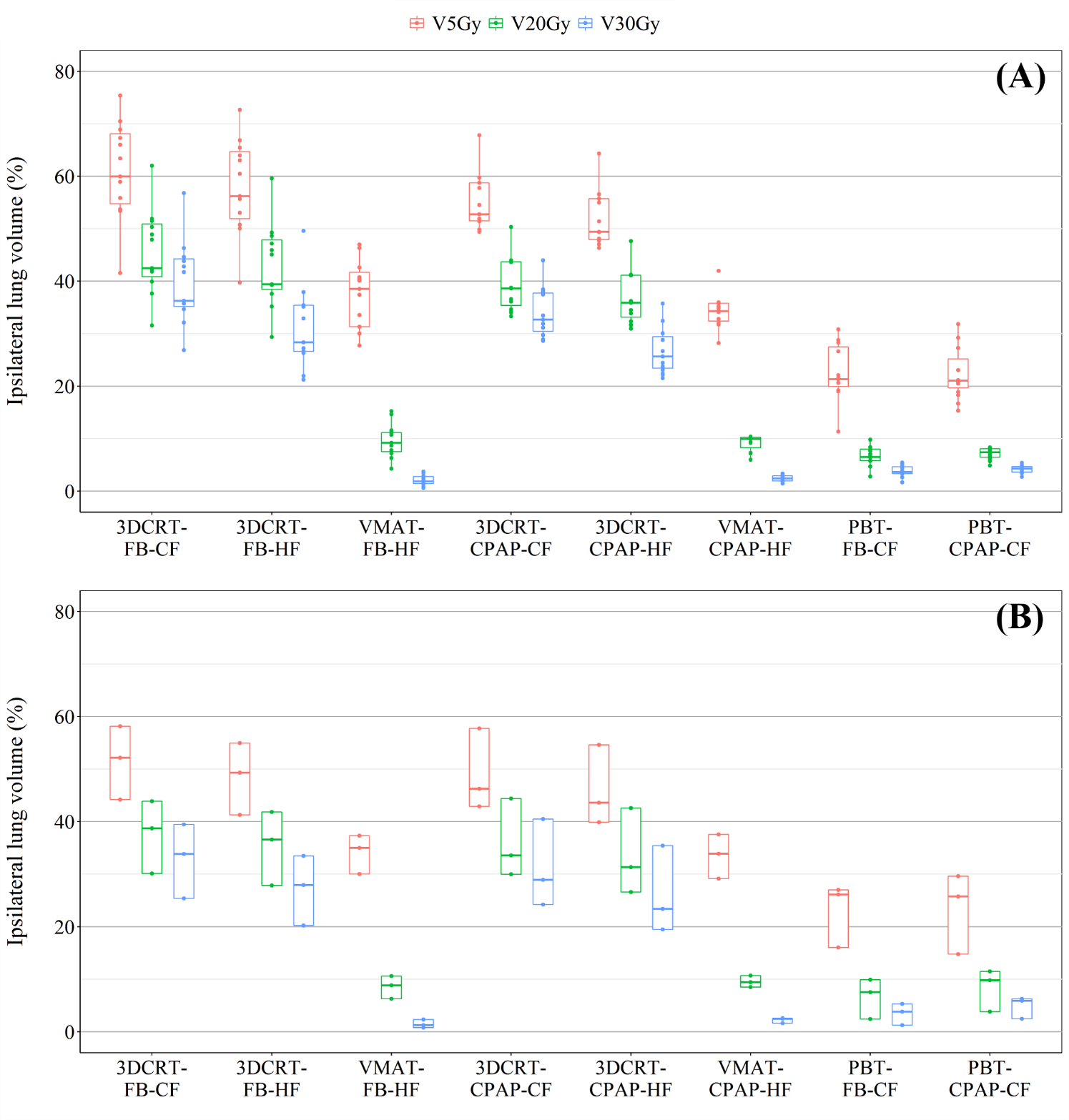


**2.4 Supplementary Figure 3.** Ipsilateral lung volumes receiving over 5, 20, and 30 Gy per patient per technique for the patients who have undergone each surgery. (A) 12 breast conservation surgery patients (B) 3 modified radical mastectomy patients


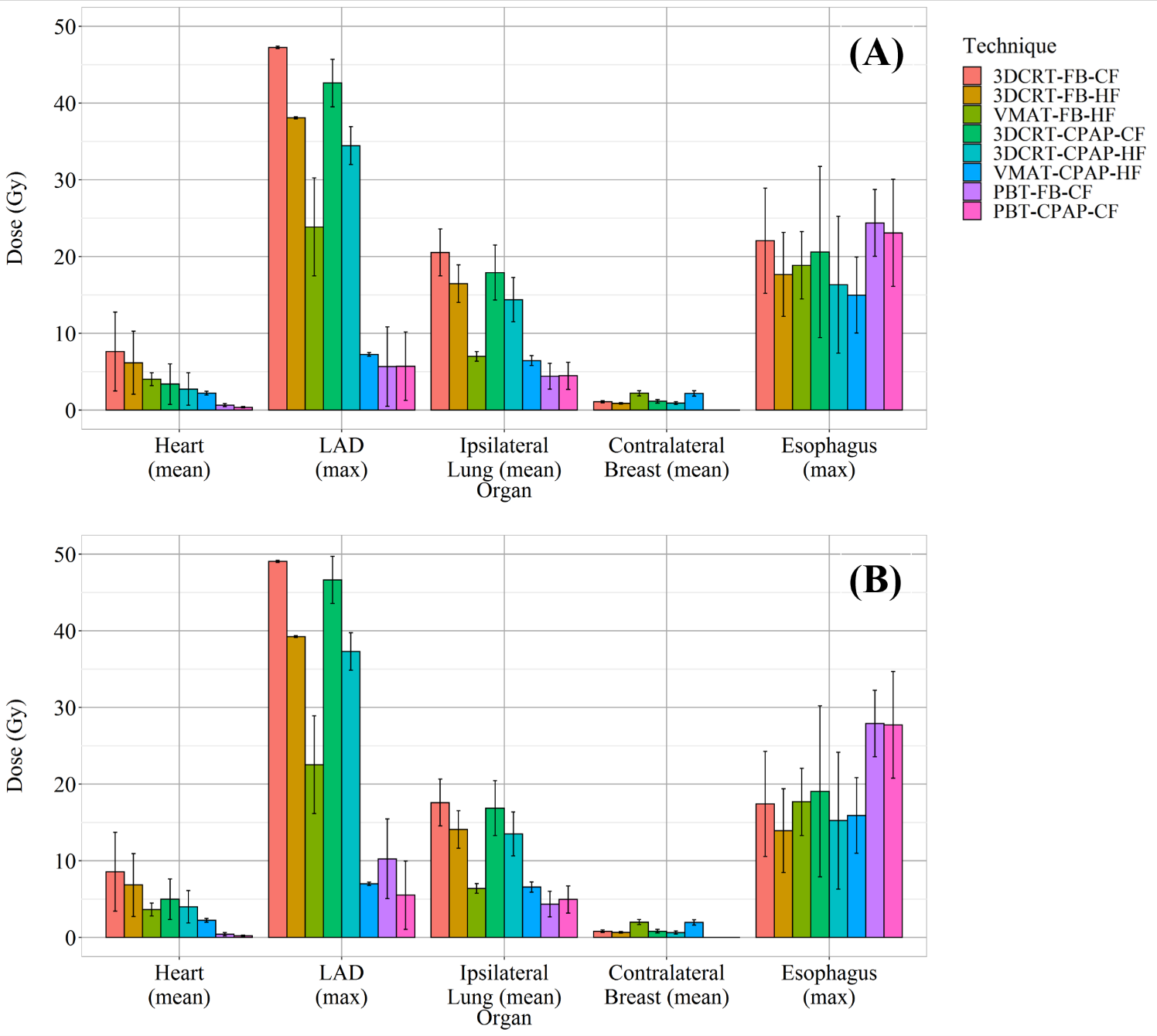

Supplement: Supplementary file 1 [file DataSheet_1.docx]
